# Age-Related Palatal Wound Healing: An Experimental In Vivo Study

**DOI:** 10.3390/biology10030240

**Published:** 2021-03-19

**Authors:** Liat Chaushu, Svetlana Atzil, Marilena Vered, Gavriel Chaushu, Shlomo Matalon, Evgeny Weinberg

**Affiliations:** 1 Department of Periodontology and Oral Implantology, The Goldschleger School of Dental Medicine, Sackler Faculty of Medicine, Tel Aviv University, Tel Aviv 69978, Israel; evgenywein@gmail.com; 2 The Goldschleger School of Dental Medicine, Sackler Faculty of Medicine, Tel Aviv University, Tel-Aviv 69978, Israel; svetuliah@gmail.com; 3 Department of Oral Pathology, Oral Medicine and Maxillofacial Imaging, The Goldschleger School of Dental Medicine, Sackler Faculty of Medicine, Tel Aviv University, Tel-Aviv 69978, Israel; lmy@netvision.net.il; 4 Department of Oral and Maxillofacial Surgery, Sackler Faculty of Medicine, The Goldschleger School of Dental Medicine, Tel Aviv University, Tel-Aviv 69978, Israel; gabi.chaushu@gmail.com; 5 Department of Oral Rehabilitation, Head, The Goldschleger School of Dental Medicine, Sackler Faculty of Medicine, Tel Aviv University, Tel-Aviv 69978, Israel; matalons@tauex.tau.ac.il; 6 Department of Oral Biology, The Goldschleger School of Dental Medicine, Sackler Faculty of Medicine, Tel Aviv University, Tel-Aviv 69978, Israel

**Keywords:** inflammation, myofibroblasts, palate, rats, wound healing

## Abstract

We assessed age-related excisional palatal mucoperiosteal wound closure in rats. A 4.2 mm diameter punch was used to create a secondary healing defect in the palate of Wistar rats. Study group—21, 18-month-old vs. control 21, 2-month-old males. The 2-dimensional area, maximum length and width of the soft tissue defect served as clinical outcome parameters. The dynamics of the initial three healing weeks were assessed. Semi-quantitative histomorphometric analysis of inflammation and myofibroblasts served for the evaluation of the inflammatory and proliferative wound healing phases. Complete wound closure was faster in the old rats. A dimensional related wound closure was observed in the young rats versus a symmetrical wound closure in the old rats. Inflammatory response was significantly delayed and of lower intensity in the old rats. Myofibroblastic response, representing the proliferative stage, was delayed and of lower intensity in the old rats, albeit not statistically significant. Reduced initial tissue damage due to decreased and delayed inflammatory response in the old rats ultimately led to faster clinical wound healing compared to the young rats, despite a statistically non-significant lower proliferative response in the old rats.

## 1. Introduction

The surgical treatment of gingival recession is a popular surgical procedure in the periodontal armamentarium, with the free soft tissue graft being one of the most popular [1]. Apart from the intra-oral applications, free gingival grafts are successfully used in several medical specialties, such as otolaryngology [2,3], ophthalmology [4], dermatology, and plastic surgery [5,6]. The palatal mucosa mesial to the first molar is ideal anatomically [7]. Graft thickness may be ensured [8] without endangering anatomical critical structures (greater palatine complex). The main disadvantages of free soft tissue grafts are donor site morbidity, such as discomfort, bleeding, pain, swelling, difficulty in chewing, eating or speaking, bad smell, infection and loss of sensation [9]. The free gingival graft (FGG) palatal wound will usually heal within 2–4 weeks [10], despite the denuded palate. The main advantage of FGG is the ease of performance and large volume of soft tissue acquired [1].

Experimental wound healing has been vastly studied [11]. It is a dynamic, interactive process involving soluble mediators, blood cells, extracellular matrix, and parenchymal cells. Wound healing has three phases—inflammation, tissue formation, and tissue remodeling—that overlap in time. The reaction pattern may differ depending on the individual and location [12]. The healing of excisional wounds in the palatal mucosa of rats has been investigated [13]. However, excisional wounds in the palate with a large soft-tissue defect are largely dependent on the individuum [14,15,16]. Secondary healing with epithelial cell migration from the periphery towards the central part of the defect is necessary to close the wound. Interaction between epithelium and connective tissue, the viability of underlying bone, inflammatory and reparative processes and many more factors are crucial [13,16].

Age-related challenges in wound healing have been suggested [17]. Experimental data have shown that palatal wound healing may be affected by aging [18]. Although most wounds heal, they have a longer duration as a result of compromises in all wound healing stages [19]. The inflammatory [18] and proliferative responses may be decreased or delayed [20]. Phases of the wound-healing process, including epithelial migration, granulation tissue formation, connective tissue formation and tissue remodeling, may be hampered [19].

The primary goal of wound treatment is rapid closure. Recent advances in cellular and molecular biology have greatly expanded our understanding of the biologic processes involved in wound repair and tissue regeneration, and may lead to improvements in wound care. Improving the treatment of the elderly is one of tomorrow’s leading challenges. The aim of the present experimental comparative study was to assess age-related differences in early palatal wound healing between the young and old in a rat model.

## 2. Materials and Methods

### 2.1. Animals and Preparation of Experimental Model

The Ethics and Institutional Animal Care and Use Committee of Tel Aviv University approved the study protocol (approval number—01-16-034). All animals received humane care.

The study design followed the Animal Research Reporting In Vivo Experiments (ARRIVE) guidelines [21]. The young group consisted of 21 Wistar-derived, 2-month-old male rats, each weighing an average of 240 g. The older group consisted of 21 Wistar-derived, 18-month-old male rats, each weighing an average of 650 g. General anesthesia was achieved with 10% Ketamine (90 mg/1 kg; Kepro, Deventer, Holland) and 2% Xylazine (10 mg/1 kg; Medical Market, Tel Izhak, Israel) injected intra-peritoneally.

A circular excisional wound 4.2 mm in diameter (i.e., initial anteroposterior (A-P) and laterolateral (L-L) dimensions) was made in the center of the palatal mucosa using a tissue punch (MIS Implant Technologies, Bar Lev Industrial Park, Israel), creating a wound area of 13.85 mm^2^ (initial wound area) as described previously [22]. These initial measurements were considered as time 0 (W0). Palatal soft tissue specimens were removed by a sharp dissection. Consequently, a circular area of denuded bone was left for secondary healing [13,23,24]. Gentle pressure was applied with gauze until hemostasis was achieved. All surgical procedures were performed by the same experienced operators (EW, GC). A 2 h postoperative break of feeding was advocated to minimize potential mechanical injury. No medications were given postoperatively to avoid any chemical effect on wound healing.

Animals were randomly sacrificed using CO_2_ inhalation at one week (W1), 2 weeks (W2) and 3 weeks (W3) post-operatively. The maxillae were separated and transferred for fixation into 10% buffered formalin for 24 h. The specimens were divided into three equal experimental groups (7 animals per group) according to the day of sacrifice.

### 2.2. Macroscopic Evaluation

**Figure 1:**
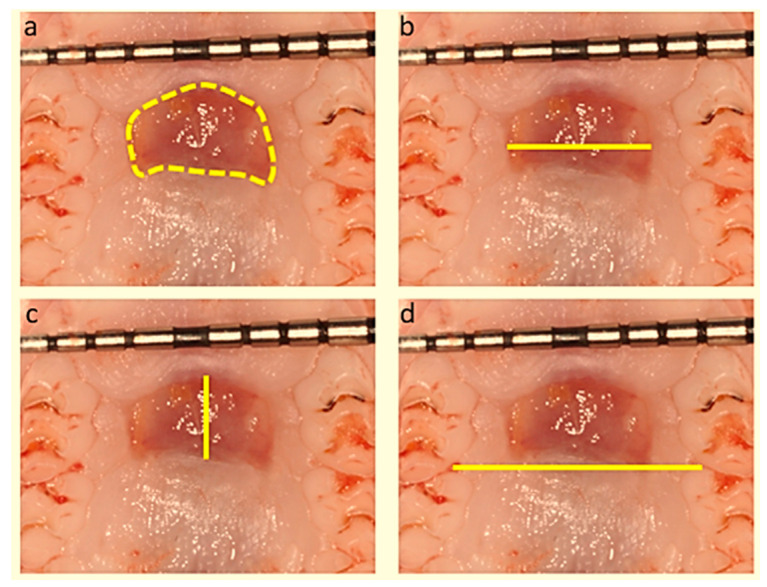
Representative photographs, using 1 mm periodontal probe as scale measurement, of the wound 14 days (W2) after surgery. The wound margins were marked (**a**) and the following measurements were taken: the final wound area (**a**), the final L-L distance (**b**), the final A-P distance (**c**) and the arch distance (**d**).

The palate specimens were photographed using a 15 mm long University of North Carolina (UNC) color-coded periodontal probe with millimeter markings (Hu Friedy Manufacturing Inc., Chicago, IL, USA) in a standardized manner using a Cannon EOS 550D camera (Canon Inc., Tokyo, Japan) immediately after tissue harvesting W0 and immediately after sacrifice prior to maxillary harvesting for histological examination W1, W2 (Figure 1) and W3.

The images were analyzed using ImageJ software (https://imagej.net/, ImageJ, RRID:SCR_003070, accessed on 28 January 2017) and calibrated with the periodontal probe markings. Digital photographs were magnified by computer and the boundaries of the wound were determined on the magnified image. The wound margins were marked and the following measurements were taken at each time point (W1, W2, W3): the total area of the wound (total wound area), the maximum L-L and the A-P dimensions of the wound. In addition, the palatal width (arch distance) was assessed as the intermaxillary distance between the first and second molar contact points (Figure 1). The mean values of these parameters were calculated and the degree of wound healing was expressed as a percentage (%) between the initial value (W0) and any of the other time point values ((W1 vs. W0), (W2 vs. W0), (W3 vs. W0)) in the same animal.

### 2.3. Microscopic Examination

After 24 h of fixation in formalin, sections were decalcified in 10% formic acid (Merck, Germany) for about 3 weeks, or until the bone had undergone sufficient decalcification to allow for sectioning.

The samples (*n* = 42) were then macroscopically cut in the frontal plane through the point of maximum L-L distance of the wound, followed by embedding in paraffin. Three micron-thick sections were prepared and stained with hematoxylin and eosin (H&E). Each of the stained slides was photographed at ×20 using a light microscope (Olympus BH-2, Tokyo, Japan), equipped with a digital camera (Olympus DP71, Tokyo, Japan).

### 2.4. Immunohistochemistry for Identification of Myofibroblasts

From each paraffin block (*n* = 42), a 3 µ-thick section was cut and mounted on positive-charged microscope slides (OptiplusTM, Biogenex, San Ramon, CA, USA), dewaxed in xylene, dehydrated in ethanol, rinsed in distilled water, placed in 3% H_2_O_2_, and rinsed again in distilled water. Antigen retrieval was performed by placing slides in citrate buffer solution, pH = 6, and heating in a microwave oven at 92 °C for 10 min. After cooling, the slides were incubated with the primary antigen for the detection of α-smooth muscle actin (α-SMA, clone 1A4, 1:100, Dako A/S, Glostrup, Denmark) for 60 min at room temperature. The universal immune peroxidase polymer anti-mouse rabbit Histofine^®^ (Multi) kit (Nichirei, Tokyo, Japan) was used for the detection of antibodies. The sections were rinsed in Phosphate Buffered Saline (PBS), reacted with an amino ethyl-carbazole substrate chromogen kit (Zymed, San Francisco, CA, USA), counterstained in Mayer’s hematoxylin (Pioneer Research Chemicals, Colchester, UK) and covered with glycerol vinyl alcohol mounting medium (Zymed, San Francisco, CA, USA). Positive control tissues comprised of a colon smooth muscle layer. Negative control was achieved by omission of the primary antibody.

### 2.5. Histomorphometry for Assessment of the Intensity of Inflammation and Density of Myofibroblasts

**Figure 2:**
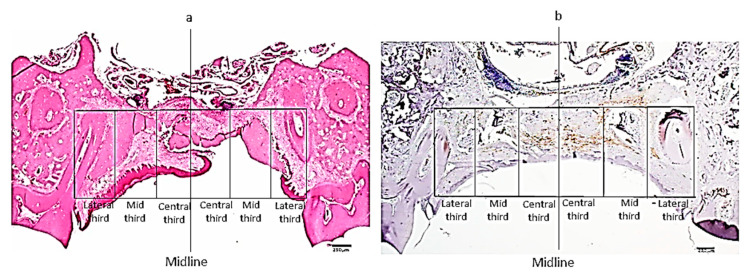
Representative photomicrographs of the wound region in which histometric measurements were made for assessing the degree of inflammation on hematoxylin and eosin-stained slides (**a**) and density of α-SMA-stained myofibroblasts (**b**), (**a**,**b**)—original magnification ×20). Scale bar = 250 µm.

H&E and α-SMA stained sections (*n* = 21, each) were photographed at ×20 using a light microscope (Olympus BH-2, Tokyo, Japan) equipped with a digital camera (Olympus DP71, Tokyo, Japan), with the original files being saved in JPEG files. Then, each file was copied on a full screen PPT slide, on which a vertical line was drawn at the midline of the palate. Two perpendicular parallel horizontal lines were drawn, encasing the area of the wound: one was between the palatal (latero-lateral) aspects of the alveolar ridges (roughly corresponding to the dentino-enamel junction of the molars), and the second was beneath the nasal mucosa (Figure 2a,b). Vertical lines parallel to the midline were drawn through the dentino-enamel junctions so that they intersected at right angles with the horizontal lines, forming a rectangle from each side of the midline (right and left). Each rectangle was further divided into 3 equal parts, thus creating “mirror” equal central, mid, and lateral rectangles, which were termed as “thirds”, as described elsewhere [22].

A semi-quantitative assessment of the intensity of the inflammatory reaction and density of myofibroblasts in each third was assessed using a 0 to 4 score system: 0 = no inflammatory/α-SMA-stained cells, 1 = a few inflammatory/α-SMA-stained dispersed cells, 2 = similar to “1” with the addition of small foci consisting of <10 cells, and 3 = similar to “2” with foci comprising >10 cells all over the examined third [25]. For each sample, there were 2 scores for each third. Results were presented as the mean score for each third at each time point.

### 2.6. Statistical Analysis

Sample size was calculated using G-Power software, based on the following assumptions: type I error (alpha) of 5% and statistical power of 80%.

Data were entered and analyzed in SPSS version 24. First, descriptive statistics were produced, while means and standard deviations were calculated for all continuous measures. Prior to main analyses, to test if main study measures distributed normally for the total sample, Kolmogorov–Smirnov tests were conducted. All measures presented normal distribution (*p* > 0.05).

For statistical analysis of the wound measurements at different time points (Week 1, 2 and 3) within two groups (young versus old rats), two-way analyses of variance (ANOVA) were used. Interactions between distance over time and group were specifically examined. A significant interaction indicates that the change in size of the wound over time depends on the age of the rats.

Mann–Whitney test was used to analyze statistical significance for histomorphometric (inflammation reaction and density of myofibroblasts) scores. Significance was reported as *p* < 0.05.

## 3. Results

**Figure 3:**
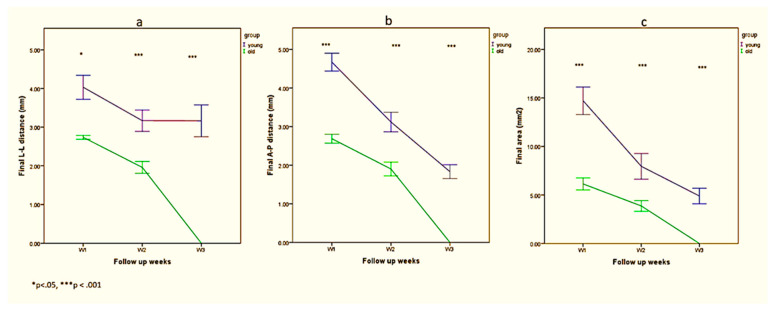
Differences between young and old rats in L-L distance (**a**), A-P distance (**b**) and total area (**c**) from week 1 to week 3; * *p* < 0.05, *** *p* < 0.001.

Table 1 and Figure 3 show differences as a factor of time in distance indicators (mm) in young and old rats.

Regarding L-L distance (Figure 3a), it was found that it was smaller among old rats in week 1 (*p* = 0.03), week 2 (*p* < 0.001), and week 3 (*p* < 0.001). A significant interaction between weeks and group was found (Table 1, *p* < 0.01), indicating that L-L distance decreased in a more rapid fashion among old rats, reaching complete healing after 3 weeks only in the old rats.

Regarding A-P distance (Figure 3b), it was found that it was smaller among old rats in weeks 1 (*p* < 0.001), 2 (*p* < 0.001), and 3 (*p* < 0.001). No significant interaction between weeks and group was found (Table 1, *p* = 0.26), indicating that A-P wound distance had a similar decreasing trend among young and old rats, reaching complete healing after 3 weeks only in the old rats.

Total wound area (Figure 3c) was smaller among old rats in weeks 1 (*p* < 0.001), 2 (*p* < 0.001), and 3 (*p* < 0.001). No significant interaction between weeks and group was found (Table 1, *p* = 0.17), indicating that total area had a similar decreasing trend among young and old rats, reaching complete healing after 3 weeks only in the old rats.

**Figure 4:**
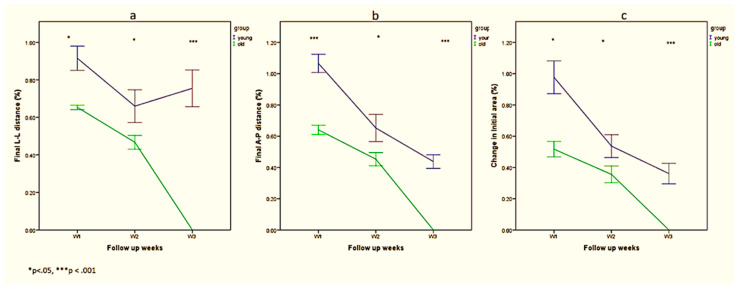
Differences between young and old rats in L-L fraction (**a**), A-P fraction (**b**) and total area fraction (**c**) relative to initial distance from week 1 to week 3; * *p* < 0.05, *** *p* < 0.001.

To test recovery by change, ANOVA analyses were conducted for changes in percentages (Table 2 and Figure 4).

In L-L dimension (Figure 4a), fraction change was higher among old rats in weeks 1 (*p* < 0.05), 2 (*p* < 0.05), and 3 (*p* < 0.001). A significant interaction between weeks and group was found (*p* < 0.01), indicating that L-L distance decreased more rapidly among old rats. No significant changes were noted in the young rats between weeks 2 and 3 (the value was 75.50 for week 3, whereas in week 2 the value was 66.00), demonstrating the delay in wound healing.

In A-P dimension (Figure 4b), change was lower among old rats in weeks 1 (*p* < 0.05), 2 (*p* < 0.05), and 3 (*p* < 0.001). After 1 week, a mean fraction of 106.25 ± 16.50% was recorded in the young rats (Table 2), indicating the amplification of the initial distance in the young rats vs. a statistically significant reduction (*p* = 0.002) in the distance, 64.67 ± 5.16%, as a result of wound healing processes in the old rats. A significant interaction between weeks and group was found (*p* = 0.04), indicating that A-P decreased more rapidly among old rats.

The change in total area fraction (Figure 4c) was higher among old rats in weeks 1 (*p* < 0.05), 2 (*p* < 0.05), and 3 (*p* < 0.001). A significant interaction between weeks and group was found (*p* = 0.02), indicating that total area decreased more rapidly among old rats.

### 3.1. Microscopic Evaluation

#### 3.1.1. Semi-Quantitative Evaluation of Inflammatory Phase

**Figure 5:**
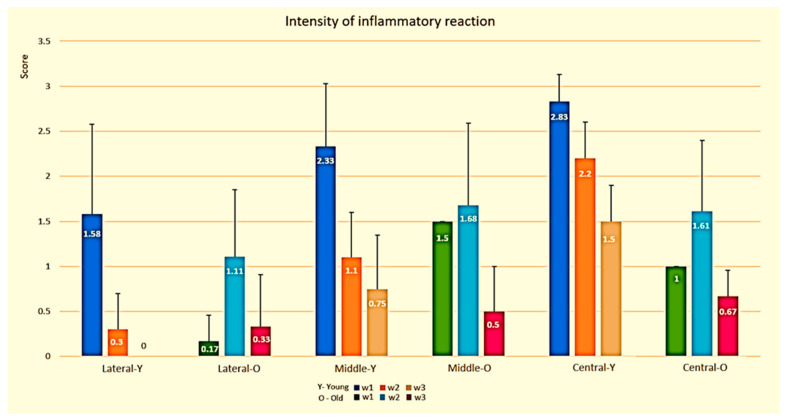
Inflammatory reaction score in the young and old rats along time.

In the young rats, the initial inflammatory reaction was high in all evaluated areas (Figure 5). In week 3, it remained high only in the middle area.

Results in the old rats (Figure 5) showed a significant increase (*p* < 0.05) from W1 to W2 in the lateral and central thirds, followed by a significant decrease (*p* < 0.05) from W2 to W3 in all thirds.

The comparison between the young and the old rats (Figure 5) demonstrated that in W1, the inflammatory response showed a higher intensity in the young rats than in the old rats in all thirds (*p* < 0.05 in the lateral and central thirds). In W2, the inflammatory response showed a higher intensity in the old rats than in the young rats in the lateral and mid thirds (*p* < 0.05 in lateral third), while it was borderline higher in the young rats than in the old rats in the central third (*p* = 0.07). In W3, the inflammatory response in young rats was borderline higher than in old rats only in the central third (*p* = 0.054).

It can be summarized that the overall inflammatory response in the old rats was delayed with a lower intensity.

#### 3.1.2. Semi-Quantitative Evaluation of the Proliferative Phase (Myofibroblasts)

**Figure 6:**
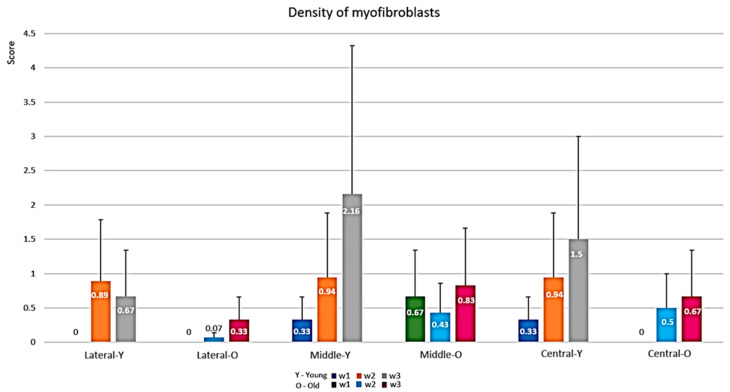
Myofibroblast density score in young and old rats along time.

The comparison of the young and the old rats (Figure 6) yielded higher values in the young rats in most areas at most time frames. However, this reached statistical significance only in the lateral third in W2. It can be inferred that the myofibroblastic response is delayed with lower intensity in the old rats; however, the differences between young and old rats were not statistically significant in most areas and at most time frames.

**Figure 7:**
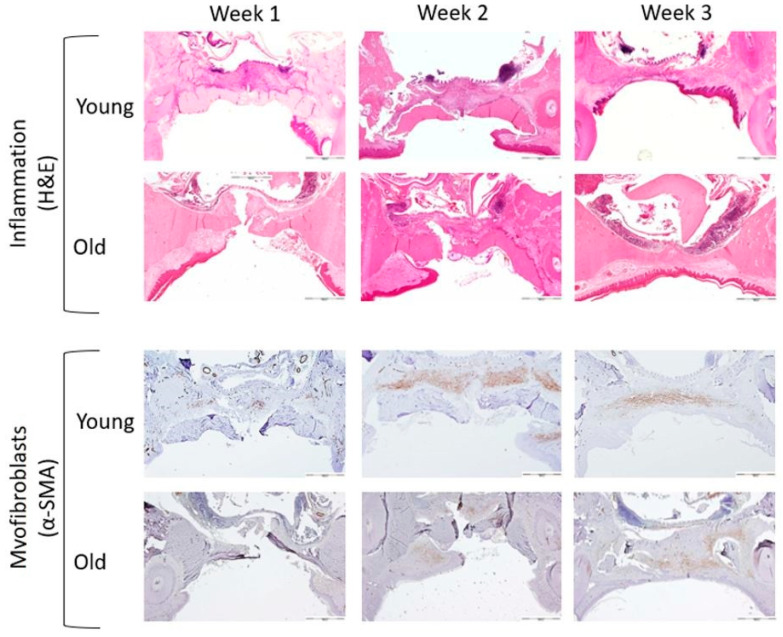
Representative photomicrographs of inflammation and myofibroblasts in young and old rats, weeks 1–3 (original magnification of all microscopic sections ×40). Scale bar 500 µm. Hematoxylin and eosin stain (H&E), Alpha Smooth Muscle Actin (α-SMA).

The overall histological comparison between the young and old rats in terms of inflammation and myofibroblasts at the various time points of the study is illustrated in Figure 7.

## 4. Discussion

Despite existing studies using experimental palatal wounds as a model, age-related data regarding the dynamics of the healing process at the wound site remain incomplete. The novel approach of the present study lies in investigating the age-related macroscopic pattern of secondary intention mucoperiosteal wound healing outcome in the A-P vs. the L-L planes.

A significant decrease was found for the A-P vs. an insignificant decrease in the L-L dimension along time in the young rats, only during the first week. A significant decrease was found for both A-P and L-L dimensions along time in the older rats. No dimensional changes were noted during the first week in the young rats vs. significant in the old rats. It can be speculated that the increased inflammatory reaction (more specifically, increased inflammatory and stem cell numbers) demonstrated in the young rats, especially in the first week, is responsible for the increased distance in each time frame as a result of additional wound damage caused by the amplified inflammatory reaction.

**Figure 8:**
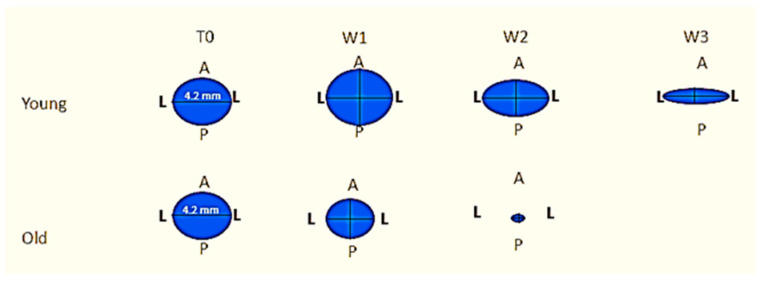
Wound closure dynamics.

The macroscopic healing (Figure 8) at W1 was minimal in the young rats, as shown previously [13,23], and significant in the old rats. As stated previously, the increased inflammatory reaction in young rats may be responsible. From W1, during the proliferative phase in both groups the defect gradually filled with soft tissue. The changes were statistically significant and more prominent in the old rats. The myofibroblastic score demonstrated no statistically significant differences between the young and the old rats. These findings suggest that the faster wound closure in the old rats cannot be attributed merely to their improved proliferative abilities. Interventions to promote wound healing should thus be used prior to the end of W2 [26].

Previous studies exist [13,23,24]. In the present study, full epithelization at W3 occurred in the old rats but not in the young rats. It can be speculated that the larger total wound area in the young rats in W1 created a worse starting point.

Periodontal disease is a good example for a similar situation [27]. In the past, evidence indicated that periodontitis is a pathogen-induced disease [28]. Nowadays, there is a shifting paradigm in the pathogenesis of periodontitis [29]. More and more studies demonstrated that periodontal disease is a result of exaggerated inflammatory reaction [27,28,29]. Consequently, a host modulation therapy (HMT) strategy was suggested [27]. The modulation of inflammatory reaction is called HMT [29]. Experimental results demonstrate encouraging results following the administration of HMT in periodontal disease [30,31]. It may be speculated that the older age in the present model acts similar to HMT agents, decreasing the inflammatory reaction and yielding an improved, faster wound healing process.

Unfortunately, a direct comparison of the current study to the untreated control groups in human studies is not feasible, since the common limitations encountered in clinical studies that evaluate donor healing, such as distinct techniques of graft harvesting, may impair the standardization of wound depth and the thickness of the graft. However, similar to the palatal donor site healing after split thickness FGG harvesting [11,26,32,33], we found that the periphery of the mucoperiosteal palatal wounds in rats filled earlier compared with the center of the wound.

Only macroscopic measurements of the dimensional changes in the wound were performed in the present study. Primarily, the margin of newly formed epithelium is not the same as that of the macroscopic wound area, while the epithelium grows over the macroscopic margin of the wound [24]. Furthermore, we found it inaccurate to microscopically determine the dimensional changes and epithelization rate of the wound due to technical factors that might have affected the accuracy of the measurements.

Study limitations must not be ignored. Rat study is not exactly like human study. The number of animals may be increased in future studies. Similarly, time frames may be increased, especially in the first week. Furthermore, conclusions should be applied in clinical situations. More specifically, using anti-inflammatory mediators preoperatively or during the first week may significantly improve wound healing in the young, whereas in the old they are less needed.

## 5. Conclusions

Collectively, our findings highlight a dynamic process of wound healing in this model. While there were minimal changes in the soft tissue L-L plane, most of the wound healing dynamics arose from the A-P plane in the young rats vs. symmetric wound closure in both dimensions in the old rats. The symmetric wound closure yielded a complete and faster wound healing. Reduced inflammatory reaction in the old rats, initially resulting in decreased tissue damage, may be responsible for the differences.

## Figures and Tables

**Table 1:** Macroscopic wound area measurements, mean and standard deviation (SD) in young and old rats from week 1 to week 3.

Measured Parameter	Young (*n* = 21)	Old (*n* = 21)	*p*	F_interaction_	*p*_interaction_
L-L distance (mm)				5.31	<0.01
Week 1	4.03 ± 0.87	2.73 ± 0.08	0.03		
Week 2	3.16 ± 0.82	1.96 ± 0.59	<0.001		
Week 3	3.18 ± 0.92	0.00 ± 0.00	<0.001		
A-P distance (mm)				1.39	0.26
Week 1	4.66 ± 2.65	2.68 ± 0.20	<0.001		
Week 2	3.12 ± 0.75	1.90 ± 0.68	<0.001		
Week 3	1.83 ± 0.45	0.00 ± 0.00	<0.001		
Total area (mm^2^)				1.84	0.17
Week 1	14.71 ± 4.01	6.13 ± 1.06	<0.001		
Week 2	7.96 ± 3.96	3.80 ± 2.13	<0.001		
Week 3	4.89 ± 1.60	0.00 ± 0.00	<0.001		

**Table 2:** Changes (%) between initial and final area, mean and standard deviation (SD) in young and old rats from week 1 to week 3 relative to W0 (4.2 mm diameter).

Measured Parameter	Young (*n* = 21)	Old (*n* = 21)	*p*	F_interaction_	*p*_interaction_
L-L fraction relative to W0 (%)				5.99	<0.01
Week 1	91.00 ± 16.70	65.33 ± 2.08	0.04		
Week 2	66.00 ± 27.55	46.73 ± 14.26	0.03		
Week 3	75.50 ± 19.53	0.00 ± 0.01	<0.001		
A-P fraction relative to W0 (%)				4.83	0.04
Week 1	106.25 ± 16.50	64.67 ± 5.16	0.002		
Week 2	65.20 ± 27.51	45.27 ± 16.36	0.032		
Week 3	43.75 ± 8.73	0.00 ± 0.01	<0.001		
Total area fraction relative to W0 (%)				3.47	0.02
Week 1	97.65 ± 29.64	51.67 ± 8.60	0.030		
Week 2	53.62 ± 21.91	35.53 ± 20.87	0.025		
Week 3	36.00 ± 13.14	0.00 ± 0.01	<0.001		

## Data Availability

The data that support the findings of this study are available from the corresponding author, [LC], upon reasonable request.
